# Semirapid Detection of Piperacillin/Tazobactam Resistance and Extended-Spectrum Resistance to β-Lactams/β-Lactamase Inhibitors in Clinical Isolates of Escherichia coli

**DOI:** 10.1128/Spectrum.00801-21

**Published:** 2021-10-20

**Authors:** Ángel Rodríguez-Villodres, Alicia Gutiérrez Linares, Lydia Gálvez-Benitez, Jerónimo Pachón, José Antonio Lepe, Younes Smani

**Affiliations:** a Clinical Unit of Infectious Diseases, Microbiology and Preventive Medicine, Hospital Universitario Virgen del Rocío, Seville, Spain; b Institute of Biomedicine of Seville (IBiS), Hospital Universitario Virgen del Rocío/CSIC/University of Seville, Seville, Spain; c Department of Medicine, University of Seville, Seville, Spain; University of Cincinnati

**Keywords:** ESRI, *Escherichia coli*, beta-lactamase, beta-lactamase inhibitor, piperacillin, resistance, tazobactam

## Abstract

Piperacillin/tazobactam (TZP) is a β-lactam/β-lactamase inhibitor (BL/BLI) recommended for the empirical treatment of severe infections. The excessive and indiscriminate use of TZP has promoted the emergence of TZP-resistant Escherichia coli isolates. Recently, we demonstrated that TZP may contribute to the development of extended-spectrum resistance to BL/BLI (ESRI) in E. coli isolates that are TZP susceptible but have low-level resistance to BL/BLI (resistance to amoxicillin/clavulanic acid [AMC] and/or ampicillin/sulbactam [SAM]). This raises the need for the development of rapid detection systems. Therefore, the objective of this study was to design and validate a method able to detect TZP resistance and ESRI in E. coli. A colorimetric assay based on β-lactam ring hydrolysis by β-lactamases was designed (ESRI test). A total of 114 E. coli isolates from bloodstream and intra-abdominal sources, characterized according to their susceptibility profiles to BL/BLI, were used. Detection of the three most frequent β-lactamases involved in BL/BLI resistance (*bla*_TEM_, *bla*_OXA-1_, and *bla*_SHV_) was performed by PCR. The ESRI test was able to detect all the TZP-intermediate/-resistant isolates, as well as all the TZP-susceptible isolates with a capacity for ESRI development. Their median times to results were 5 and 30 min, respectively. All the isolates without resistance to BL/BLI displayed a negative result in the ESRI test. *bla*_TEM_ was the most frequent β-lactamase gene detected, follow by *bla*_SHV_ and *bla*_OXA-1_. These results demonstrate the efficacy of the ESRI test, showing great clinical potential which could lead to reductions in health costs, ineffective treatments, and inappropriate use of BL/BLI.

**IMPORTANCE** TZP is a BL/BLI recommended for the empirical treatment of severe infections. The excessive use of TZP has promoted the emergence of TZP-resistant Escherichia coli isolates. We recently reported that TZP may contribute to the development of ESRI in E. coli isolates that are TZP susceptible but have low-level resistance to BL/BLI. This raises the need for the development of rapid detection systems. Here, we demonstrated that the ESRI test was able to detect the TZP-intermediate or -resistant isolates and the TZP-susceptible isolates with the capacity for ESRI development. All the isolates without BL/BLI resistance were negative for the ESRI test and did not harbor β-lactamase genes. For ESRI developers and TZP-intermediate or -resistant isolates, *bla*_TEM_ was the most frequent β-lactamase gene detected, follow by *bla*_SHV_ and *bla*_OXA-1_. The sensitivity, specificity, and positive and negative predictive values were all 100%. These data demonstrate the efficacy of the ESRI test and show that it has great clinical potential.

## INTRODUCTION

Excessive and indiscriminate use of antibiotics has accelerated the emergence and spread of antimicrobial resistance and increased the inefficacy of available antimicrobial treatments. Within the wide range of bacterial species able to produce infection in humans, the order *Enterobacterales* holds a prominent place, with Escherichia coli being the species with the highest clinical relevance (1). Among the antimicrobials used for the treatment of infections by E. coli, we found a group of β-lactams combined with β-lactamase inhibitors (BL/BLI), including ampicillin/sulbactam (SAM), amoxicillin/clavulanic acid (AMC), and piperacillin/tazobactam (TZP). The latter is efficacious against E. coli and other Gram-negative bacteria producing β-lactamases (mainly TEM enzymes) (2).

TZP is used especially in severe infections and health care-associated infections (3, 4). However, the abusive use of this antibiotic has led to the appearance of resistant strains (5, 6).

In Spain, data extracted from the Study for Monitoring Antimicrobial Resistance Trends (SMART) from 2002 to 2010 and 2016 to 2017 revealed an increase in the rate of TZP resistance in intra-abdominal E. coli isolates, from 7.7% to 10.0% (7, 8). A similar increase was observed in England between 2011 and 2015 in bacteremic E. coli isolates (9).

The presence of TEM, inhibitor-resistant TEM (IRT), OXA-1, SHV, and/or AmpC β-lactamases is a common cause of E. coli resistance to BL/BLI (10–14). Of these, TEM-1 hyperproduction and IRT are the most frequent mechanisms involved in the resistance to BL/BLI, especially to SAM and AMC and, in some cases, to TZP (15, 16).

**FIG 1:**
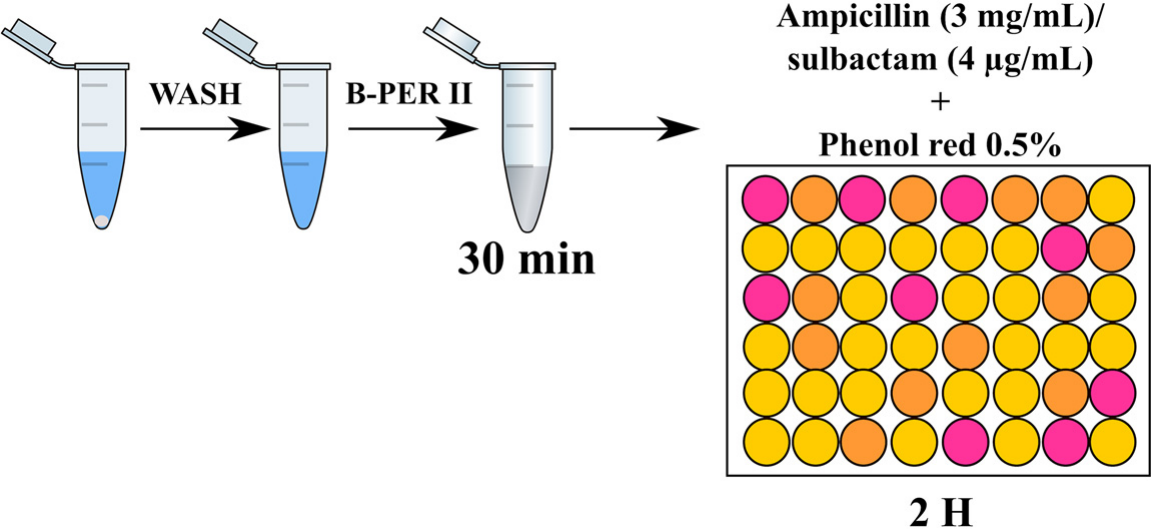
Design and protocol of the ESRI test. B-PER II, bacterial protein extraction reagent.

In E. coli, the pattern of BL/BLI resistance is a gradual and unidirectional process that extends from SAM, to TZP, through AMC (17). Thus, one isolate may be resistant to SAM but susceptible to AMC and TZP (RSS phenotype), resistant to SAM and AMC but susceptible to TZP (RRS phenotype), or resistant to all three combinations (RRR phenotype) (17). Recently, we demonstrated both *in vitro* and in patients with intra-abdominal infections that E. coli isolates with the RSS or RRS phenotype, classified as having a low level of resistance to BL/BLI, and carrying the *bla*_TEM_ gene, are able to acquire stable resistance to TZP and to the other two combinations if they are exposed to TZP at sub-MIC levels, indicating that extended-spectrum resistance to BL/BLI (ESRI), from SAM (RSS) to TZP (RRR), can be developed (17). Early detection of both resistance to TZP and the ability to develop ESRI in E. coli isolates with low-level resistance to BL/BLI (RSS or RRS) is essential to establish adequate initial and early antimicrobial treatment of severe infections, the moment in which the TZP treatment has special relevance. In clinical practice, when E. coli is isolated in a clinical sample, the Microbiology Service of hospital system reports only the susceptibility or resistance to the different BL/BLI, among other antimicrobials, but not the potential development of resistance to those agents. Therefore, there is no system that allows us to detect this possible ESRI when E. coli isolates present low-level resistance to BL/BLI (RSS or RRS). In this study, we developed a semirapid detection system to detect resistance to TZP and the potential development of ESRI in clinical isolates of E. coli (Fig. 1).

## RESULTS

### Antimicrobial susceptibility in clinical isolates of E. coli.

Susceptibility profiles to SAM, AMC, and TZP obtained through MicroScan were analyzed, allowing the selection of 114 E. coli clinical isolates with different profiles (susceptible to all three drug combinations [SSS], RSS, RRS, and RRR) in order to develop and validate the ESRI test (Tables 1 to 3). On the other hand, the TZP MICs obtained by broth microdilution classified 67 (58.7%) of the 114 E. coli isolates as susceptible, 6 (5.2%) as intermediate, and 41 (35.9%) as resistant to TZP, according to the breakpoint established by CLSI (18). The use of MicroScan and the standard broth microdilution method showed the same TZP susceptibility profiles for the majority of clinical isolates. However, some exceptions were observed: the C1-8 (RRR) and C1-102 (RRR) isolates presented MICs of 4 mg/liter and were classified as susceptible (Table 2), while the C1-136 (RRS), C1-166 (RSS), and C1-189 (RSS) isolates showed MICs of >512, >512, and 128 mg/liter, respectively, and were classified as resistant (Table 3).

**TABLE 1:** Clinical isolates of Escherichia coli that were non-ESRI developers and had negative results in the ESRI test

Isolate (*n* = 22)	Origin	BL/BLI resistance profile^*a*^	TZP MIC (mg/liter)^*b*^	Resistance mechanism^*c*^	ESRI test^*d*^				TZP pressure test^*d*^	New TZP MIC (mg/liter)
					Result at 6–8 h of growth	Time to result (min)	Result at 24 h of growth	Time to result (min)		
C1-74	Blood	RRS	16	Neg	−	>120	−	>120	−	32
C1-83	Blood	RRS	8	Neg	−	>120	−	>120	−	8
C1-90	Blood	SSS	2	Neg	−	>120	−	>120	−	2
C1-95	Blood	SSS	2	Neg	−	>120	−	>120	−	8
C1-97	Blood	SSS	4	Neg	−	>120	−	>120	−	16
C1-100	Blood	SSS	2–4	Neg	−	>120	−	>120	−	4
C1-110	Blood	SSS	4	Neg	−	>120	−	>120	−	4
C1-111	Blood	SSS	2–4	Neg	−	>120	−	>120	−	16
C1-128	Blood	SSS	2	Neg	−	>120	−	>120	−	8
C1-138	Blood	RSS	4	Neg	−	>120	−	>120	−	4
C1-144	Blood	SSS	4	Neg	−	>120	−	>120	−	4
C1-146	Blood	SSS	2–4	Neg	−	>120	−	>120	−	4
C1-152	Blood	SSS	4	Neg	−	>120	−	>120	−	4
C1-155	Blood	SSS	4	Neg	−	>120	−	>120	−	4
C1-157	Blood	SSS	2	Neg	−	>120	−	>120	−	2
C1-168	Blood	SSS	4	Neg	−	>120	−	>120	−	4
C1-169	Blood	SSS	2	Neg	−	>120	−	>120	−	2
C1-172	Blood	SSS	2	Neg	−	>120	−	>120	−	2
C1-177	Blood	SSS	4	Neg	−	>120	−	>120	−	4
C2-4	Bile	SSS	2	Neg	−	>120	−	>120	−	2
C2-8	Bile	SSS	8	Neg	−	>120	−	>120	−	8
C2-12	Bile	RRS	4	Neg	−	>120	−	>120	−	4

a SSS, susceptible to ampicillin/sulbactam, amoxicillin/clavulanic acid, and piperacillin/tazobactam; RSS, resistant to ampicillin/sulbactam and susceptible to amoxicillin/clavulanic acid and piperacillin/tazobactam; RRS, resistant to ampicillin/sulbactam and amoxicillin/clavulanic acid and susceptible to piperacillin/tazobactam.

b TZP, piperacillin/tazobactam.

c Neg, negative result in relation to studied resistance mechanisms (TEM type, OXA-1 type, and SHV type).

d −, negative result in ESRI detection or antimicrobial pressure test.

**TABLE 2:** Clinical isolates of Escherichia coli that were ESRI developers and had positive results in the ESRI test

Isolate (*n* = 45)	Origin	BL/BLI resistance profile^*a*^	TZP MIC (mg/liter)^*b*^	Resistance mechanism^*c*^	ESRI test^*d*^				TZP pressure test^*d*^	New TZP MIC (mg/liter)
					Result at 6–8 h of growth	Time to result (min)	Result at 24 h of growth	Time to result (min)		
C1-8	Blood	RRR	4	Neg	+	25	+	60	+	256
C1-31	Blood	RRS	8	TEM-40	+	25	+	60	+	64
C1-38	Blood	RRS	16	TEM-35	+	1	+	1	+	256
C1-48	Blood	RRS	4	TEM-1	+	20	+	30	+	128
C1-65	Blood	RRS	8	TEM-1	+	60	+	60	+	512
C1-72	Blood	RRS	16	TEM-1	+	30	+	60	+	512
C1-81	Blood	RRS	16	TEM-30	+	2	+	1	+	512
C1-91	Blood	RSS	1	Neg	+	40	+	20	+	256
C1-93	Blood	RSS	2	SHV	+	20	+	10	+	512
C1-99	Blood	RSS	8	TEM-1	+	40	+	30	+	>512
C1-102	Blood	RRR	4	TEM-1	+	1	+	2	+	32
C1-103	Blood	RSS	8	TEM-1	+	40	+	100	+	512
C1-106	Blood	RSS	8	TEM-1	+	40	+	25	+	512
C1-108	Blood	RSS	2	TEM-1	+	40	+	30	+	512
C1-118	Blood	RRS	16	OXA-1	+	30	+	10	+	256
C1-120	Blood	RSS	8	TEM-1	+	40	+	20	+	>512
C1-121	Blood	RSS	8	TEM-1/SHV	+	30	+	30	+	256
C1-126	Blood	RSS	8	TEM-1	+	30	+	30	+	512
C1-129	Blood	RSS	16	TEM-1	+	40	+	25	+	256
C1-130	Blood	RSS	8	TEM-1	+	40	+	25	+	256
C1-137	Blood	RSS	8	TEM-1	+	40	+	25	+	>512
C1-139	Blood	RSS	8	TEM-1	+	30	+	120	+	>512
C1-141	Blood	RSS	4	TEM-1/SHV	+	30	+	10	+	>512
C1-142	Blood	RRS	4	TEM-135/SHV	+	10	+	10	+	512
C1-143	Blood	RSS	8	TEM-1	+	30	+	60	+	>512
C1-153	Blood	RSS	8	TEM-1/SHV	+	50	+	90	+	>512
C1-159	Blood	RSS	2	TEM-1	+	100	+	30	+	512
C1-160	Blood	RSS	4	TEM-1	+	50	+	60	+	512
C1-161	Blood	RSS	16	TEM-1	+	100	+	60	+	>512
C1-162	Blood	RSS	4	Neg	+	30	+	30	+	512
C1-164	Blood	RSS	8	TEM-1	+	30	+	30	+	512
C1-170	Blood	RSS	4	TEM-1	+	30	+	60	+	>512
C1-171	Blood	RSS	8	Neg	−	>120	+	40	+	512
C1-174	Blood	SSS	2	TEM-1	+	120	+	120	+	>512
C1-175	Blood	RSS	16	TEM-1	+	30	+	60	+	512
C1-183	Blood	RSS	8	TEM-1	+	30	+	40	+	512
C1-187	Blood	RSS	2	TEM-1	+	100	+	40	+	>256
C1-190	Blood	RSS	16	SHV	−	>120	+	120	+	512
C1-191	Blood	RSS	8	TEM-1	+	60	+	100	+	256
C1-306	Blood	RSS	16	TEM-1	+	30	+	30	+	128
C2-14	Bile	RRS	8	TEM-1	+	10	+	10	+	512
C2-47	Intra-abdominal abscess	RSS	8	TEM-1	+	20	+	30	+	512
C2-49	Peritoneal fluid	RSS	8	TEM-1	+	15	+	30	+	512
C2-54	Intra-abdominal abscess	RRS	8	TEM-1	+	15	+	30	+	256
PT3	Blood	RSS	8	TEM-1	+	30	+	50	+	256 (PT4)

a SSS, susceptible to ampicillin/sulbactam, amoxicillin/clavulanic acid, and piperacillin/tazobactam; RSS, resistant to ampicillin/sulbactam and susceptible to amoxicillin/clavulanic acid and piperacillin/tazobactam; RRS, resistant to ampicillin/sulbactam and amoxicillin/clavulanic acid and susceptible to piperacillin/tazobactam; RRR, resistant to ampicillin/sulbactam, amoxicillin/clavulanic acid, and piperacillin/tazobactam.

b TZP, piperacillin/tazobactam.

c Neg, negative result in relation to studied resistance mechanisms (TEM type, OXA-1 type, and SHV type).

d −, negative result in ESRI detection or antimicrobial pressure test; +, positive result in ESRI detection or antimicrobial pressure test.

**TABLE 3:** Clinical isolates of Escherichia coli that were intermediate or resistant to TZP and had positive results in the ESRI test

Isolate (*n* = 47)	Origin	BL/BLI resistance profile^*a*^	TZP MIC (mg/liter)^*b*^	Resistance mechanism^*c*^	ESRI test^*d*^			
					Result at 6–8 h of growth	Time to result (min)	Result at 24 h of growth	Time to result (min)
C1-23	Blood	RRR	>256	TEM-1	+	2	+	10
C1-82	Blood	RRS	32	TEM-1	+	10	+	10
C1-94	Blood	RRS	32	TEM-1	+	15	+	10
C1-109	Blood	RRR	>256	SHV	+	2	+	2
C1-116	Blood	RRR	256	TEM-1	+	5	+	4
C1-136	Blood	RRS	>512	TEM-1/SHV	+	20	+	25
C1-166	Blood	RSS	>512	TEM-1	+	100	+	30
C1-189	Blood	RSS	128	TEM-1	+	10	+	10
C1-239	Blood	RRR	>256	TEM-1	+	2	+	2
C1-436	Blood	RRR	>256	TEM-1	+	3	+	3
C2-23	Bile	RRR	>256	TEM-1	+	10	+	10
C2-45	Peritoneal fluid	RRR	256	TEM-1	+	10	+	60
C2-48	Intra-abdominal abscess	RRR	>256	TEM-1	+	2	+	10
C2-57	Bile	RRR	>256	Neg	+	2	+	10
C2-72	Intra-abdominal abscess	RRR	>256	TEM-1	+	20	+	4
C2-74	Peritoneal fluid	RRR	256	TEM-1	+	5	+	3
C2-82	Hepatic abscess	RRR	>256	TEM-1/SHV	+	3	+	3
C2-90	Peritoneal fluid	RRR	256	TEM-1	+	4	+	2
C2-95	Peritoneal fluid	RRR	64	TEM-1	+	15	+	15
C2-103	Peritoneal fluid	RRR	256	TEM-1	+	2	+	3
C2-106	Peritoneal fluid	RRR	128	OXA-1	+	30	+	25
C2-113	Intra-abdominal abscess	RRR	256	TEM-1/SHV	+	2	+	3
C2-116	Intra-abdominal abscess	RRR	256	TEM-1	+	2	+	3
C2-117	Peritoneal fluid	RRR	128	TEM-12	+	5	+	3
C2-136	Peritoneal fluid	RRR	128	TEM-84	+	5	+	1
C2-146	Peritoneal fluid	RRR	>256	TEM-1	+	1	+	1
C2-147	Intra-abdominal abscess	RRR	>256	TEM-1	+	3	+	1
PT4	Blood	RRR	256	TEM-1	+	10	+	15
PTR1	Blood	RRR	64	OXA-1	+	10	+	5
PTR2	Blood	RRR	256	TEM-1	+	10	+	1
PTR3	Blood	RRR	256	Neg	+	10	+	1
PTR4	Blood	RRR	>256	Neg	+	3	+	1
PTR5	Blood	RRR	>256	TEM-1	+	3	+	1
PTR7	Blood	RRR	256	Neg	+	15	+	1
PTR8	Blood	RRR	128	OXA-1	+	30	+	2
PTR9	Blood	RRR	128	TEM-1/OXA-1	+	3	+	5
PTR10	Blood	RRR	>256	SHV	+	1	+	1
PTR11	Blood	RRR	128	TEM-1	+	3	+	1
PTR12	Blood	RRR	256	Neg	+	3	+	1
PTR13	Blood	RRR	256	TEM-1	+	3	+	10
PTR14	Blood	RRR	32	Neg	+	50	+	40
PTR15	Blood	RRR	128	OXA-1	+	10	+	10
PTR16	Blood	RRR	128	TEM-1	+	15	+	15
PTR17	Blood	RRR	>256	TEM-1/OXA-1	+	10	+	3
PTR18	Blood	RRR	128	TEM-1	+	10	+	10
PTR19	Blood	RRR	64	OXA-1	+	10	+	10
PTR20	Blood	RRR	>256	TEM-1	+	10	+	3

a RSS, resistant to resistant to ampicillin/sulbactam and susceptible to amoxicillin/clavulanic acid and piperacillin/tazobactam; RRS, resistant to ampicillin/sulbactam and amoxicillin/clavulanic acid and susceptible to piperacillin/tazobactam; RRR, resistant to ampicillin/sulbactam, amoxicillin/clavulanic acid, and piperacillin/tazobactam.

b TZP, piperacillin/tazobactam.

c Neg, negative result in relation to studied resistance mechanisms (TEM type, OXA-1 type, and SHV type).

d +, positive result in ESRI detection test.

### Detection of ESRI.

The 67 isolates classified as susceptible to TZP were exposed to TZP pressure in order to reveal any ability to develop ESRI. Of these, 22 (32.8%) did not develop ESRI (Table 1), maintaining their TZP MICs or increasing their MICs slightly but without becoming resistant. Of the remaining isolates, 45 (67.2%) were able to develop ESRI, increasing their MICs to TZP at least 8-fold (Table 2).

### Detection and sequencing of β-lactamases.

None of the *bla*_TEM_, *bla*_OXA-1_, and *bla*_SHV_ genes were detected in the 22 isolates that were susceptible to TZP and without the ability to develop ESRI (Table 1). However, of the 45 isolates susceptible to TZP and with the ability to develop ESRI, *bla*_TEM_, *bla*_OXA-1_, and *bla*_SHV_ genes were detected in 38 (84.4%), 1 (2.2%), and 6 (13.3%) isolates, respectively. Simultaneous detection of *bla*_TEM_ and *bla*_SHV_ was found in four isolates (8.8%). However, there were two E. coli isolates with the ability to develop ESRI (4.4%) in which *bla*_TEM_, *bla*_OXA-1_, and *bla*_SHV_ were not detected. Regarding the type of *bla*_TEM_ detected, 34 isolates expressed TEM-1 and the other 4 isolates each expressed TEM-30, TEM-35, TEM-40, or TEM-135 (Table 2).

In the case of the 47 isolates that were intermediate or resistant to TZP, *bla*_TEM_, *bla*_OXA-1_, and *bla*_SHV_ genes were detected in 34 (72.3%), 7 (14.9%), and 5 (10.6%) isolates, respectively. *bla*_TEM_ was found together with *bla*_SHV_ in three isolates and with *bla*_OXA-1_ in two other isolates. There were six isolates (12.7%) in which *bla*_TEM_, *bla*_OXA-1_, and *bla*_SHV_ were not detected. All the *bla*_TEM_ genes detected expressed TEM-1, except for isolates C2-117 and C2-136, which expressed TEM-12 and TEM-84, respectively (Table 3).

### ESRI test validation.

All the isolates that were susceptible to TZP and without an ability to develop ESRI presented with a negative result in the ESRI test (Table 1). Regarding the isolates susceptible to TZP and potentially ESRI developers, 43 isolates (95.5%) gave a positive result for the ESRI test at 6 to 8 h of bacterial growth. The two isolates that gave a negative result for the ESRI test became positive at 24 h of bacterial growth (Table 2). Similarly, the isolates that were intermediate/resistant to TZP were 100% positive in the ESRI test, both at 6 to 8 and 24 h of bacterial growth (Table 3).

**FIG 2:**
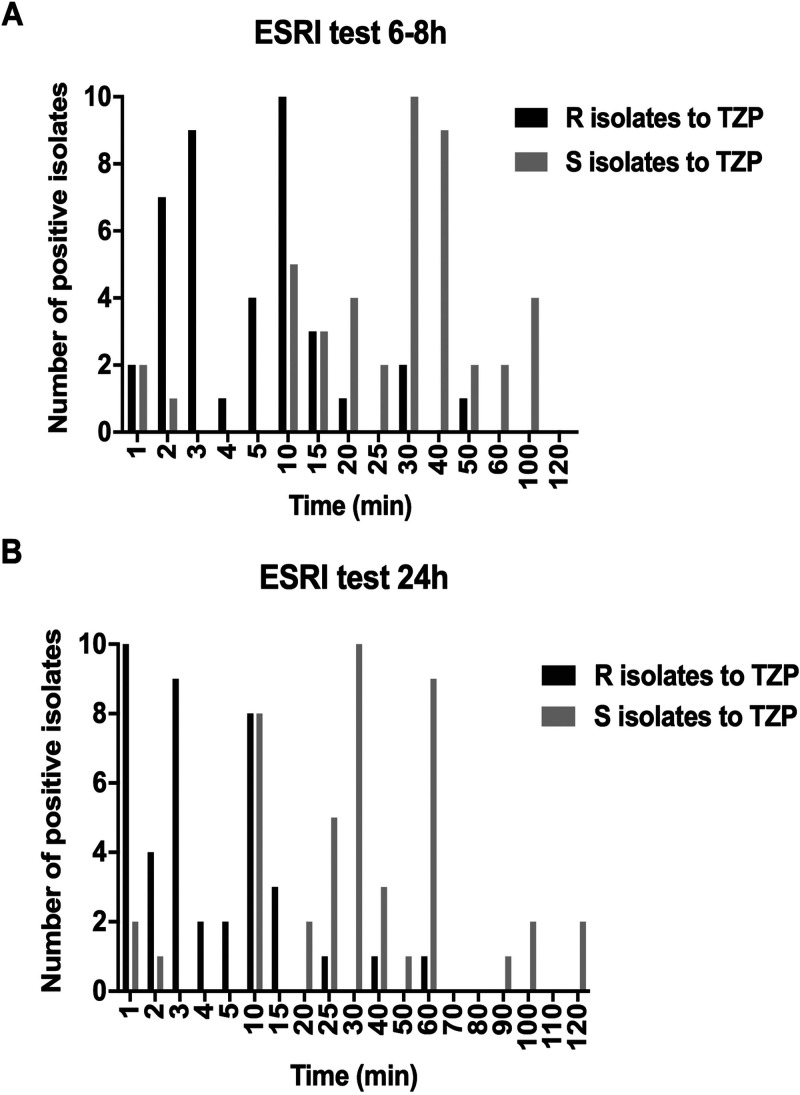
Times of ESRI test positivity in TZP-resistant and ESRI developer E. coli isolates. S, susceptible; R, resistant; TZP, piperacillin/tazobactam.

Statistically significant differences between the two groups (ESRI developer isolates and TZP-intermediate/-resistant isolates) were found in the times for positivity of the ESRI test. In the first group, the median times and interquartile ranges (IQRs) were 30 min (25 to 40 min) at 6 to 8 h of growth and 30 min (25 to 60 min) at 24 h of growth. However, in the second group, the median times and IQRs were 5 min (3 to 10 min) at 6 to 8 h (*P < *0.001) and 3 min (2 to 10 min) at 24 h (*P < *0.001) (Fig. 2). The sensitivity, specificity, positive predictive value (PPV), and negative predictive value (NPV) for the detection of isolates intermediate or resistant to TZP were 100% at both 6 to 8 h and 24 h of growth (Table 4). However, in the group of ESRI developers, the sensitivity and NPV decreased to 96% and 92%, respectively, at 6 to 8 h (Table 5).

**TABLE 4:** Validation of the ESRI test in the detection of TZP-resistant isolates (*n* = 69)

Growth (h) prior to test	No. of isolates whose ESRI results matched their phenotypic profiles/no. of isolates with phenotypic profile (%)^*a*^			
	Sensitivity	Specificity	PPV	NPV
6–8	47/47 (100)	22/22 (100)	47/47 (100)	22/22 (100)
24	47/47 (100)	22/22 (100)	47/47 (100)	22/22 (100)

a *n* = 69 isolates, corresponding to a total of 22 and 47 isolates susceptible and resistant to TZP, respectively. PPV, positive predictive value; NPV, negative predictive value.

**TABLE 5:** Validation of the ESRI test in the detection of ESRI developer isolates (*n* = 67)

Growth (h) prior to test	No. of isolates whose ESRI results matched their phenotypic profile/no. of isolates with phenotypic profile (%)^*a*^			
	Sensitivity	Specificity	PPV	NPV
6–8	43/43 + 2 (96)	22/22 (100)	45/45 (100)	22/22 + 2 (92)
24	45/45 (100)	22/22 (100)	45/45 (100)	22/22 (100)

a *n* = 67 isolates, corresponding to a total of 22 and 45 isolates susceptible to TZP and developers of ESRI, respectively. PPV, positive predictive value; NPV, negative predictive value; + 2, 2 isolates negative for ESRI test at 6–8 h.

Regarding the initial E. coli isolates, we have calculated the sensitivity and specificity of the ESRI test based on the initial resistance patterns (RSS, RRS, and RRR). The results of this analysis show a global sensitivity and specificity of 97.8% and 94.7%, respectively.

### ESRI detection in paired isolates of E. coli.

In a previous study (17), we analyzed the clinical and microbiological data from two E. coli clinical isolates (C2-49 and C2-54) belonging to sequence type 69 (ST69) and recovered from intra-abdominal abscesses of the same patient before and after 10 days of TZP treatment (4 g/0.5 g each 8 h) without clinical improvement. Similarly, we analyzed two other E. coli clinical isolates (PT3 and PT4) belonging to ST88 and recovered from blood cultures in a second patient with a perianal abscess and persistent bacteremia before and after 8 days of TZP treatment (4 g/0.5 g each 8 h) without clinical improvement. Source control was performed surgically for both patients after the first positive culture. The first isolates from both patients, C2-49 and PT3, presented an RSS resistance pattern to BL/BLI and were ESRI positive, and the second isolates, C2-54 and PT4, presented with RRS and RRR resistance patterns, respectively, and were ESRI positive (Table 6).

**TABLE 6:** Paired isolates with positive results in ESRI test

solate (*n* = 4)	Origin	ST	BL/BLI resistance profile^*a*^	TZP MIC (mg/liter)^*b*^	Resistance mechanism	ESRI test^*c*^				TZP pressure test^*c*^	New TZP MIC (mg/liter)
						Result at 6–8 h growth	Time to result (min)	Result at 24 h growth	Time to result (min)		
C2-49	Peritoneal fluid	ST69	RSS	8	TEM-1	+	15	+	30	+	512
C2-54	Intra-abdominal abscess	ST68	RRS	8	TEM-1	+	15	+	30	+	256
PT3	Blood	ST88	RSS	8	TEM-1	+	30	+	50	+	256 (PT4)
PT4	Blood	ST88	RRR	256	TEM-1	+	10	+	15	NP^*d*^	NP

a RSS, resistant to ampicillin/sulbactam and susceptible to amoxicillin/clavulanic acid and piperacillin/tazobactam; RRS, resistant to ampicillin/sulbactam and amoxicillin/clavulanic acid and susceptible to piperacillin/tazobactam; RRR, resistant to ampicillin/sulbactam, amoxicillin/clavulanic acid, and piperacillin/tazobactam.

b TZP, piperacillin/tazobactam.

c +, positive result in ESRI detection or antimicrobial pressure test.

d NP, not performed.

## DISCUSSION

In the present study, we designed a semirapid detection system, called the ESRI test, for detecting TZP resistance or the ability to develop ESRI in E. coli. We characterized a collection of 114 clinical isolates of E. coli that were used to validate the ESRI test.

Discordant results in TZP MICs between MicroScan and standard broth microdilution were observed for five (4.3%) of the E. coli isolates studied. Two replicates were performed and showed the same results. This difference could be due to technical problems associated with the development of the MicroScan method.

This study showed that *bla*_TEM_ was the most frequently detected β-lactamase gene, both in TZP-resistant and ESRI developer E. coli isolates. In contrast, *bla*_OXA-1_ was the least frequently detected β-lactamase gene in both groups, with a clear difference between TZP-resistant (14.9%) and ESRI developer (2.2%) isolates. Similar data were observed in another study, in which OXA-1 was strongly associated with TZP resistance in E. coli (13). Moreover, in some isolates, none of the studied β-lactamases were detected, probably indicating the involvement of other β-lactamases, such as AmpC (19).

It is noteworthy that the ESRI test is very sensitive and specific and has high PPV and NPV, being able to detect all of the TZP-resistant and nearly all of the ESRI developer E. coli isolates analyzed in this study. Only two ESRI developer isolates were not detected by the ESRI test at 6 to 8 h. One possible explanation is that the growth rates of both isolates were lower than those of the rest of the isolates. Another possibility is that both isolates did not produce high enough quantities of β-lactamase to be detected by the test at 6 to 8 h of bacterial growth. More studies are needed to confirm this hypothesis.

Other similar tests have been developed and/or commercialized for the detection of extended spectrum β-lactamases (ESBL) or carbapenemases, such as the ESBL NDP test (20), the Rapidec Carba NP test (21), the Nitro-Carba test (22), and matrix-assisted laser desorption ionization–time of flight mass spectrometry (MALDI-TOF MS) (23), among others. In addition, manual and automatic methods like rapid antimicrobial susceptibility testing (RAST) and the automated commercial laser-scattering-based *in vitro* system Alfred 60AST have been developed to identify TZP-resistant E. coli from positive blood culture bottles within 4 to 6 h (24, 25). The main difference from these interesting tests is that the ESRI test was designed to detect not only TZP-resistant isolates but also ESRI developer isolates, which are primarily susceptible to TZP.

**FIG 3:**
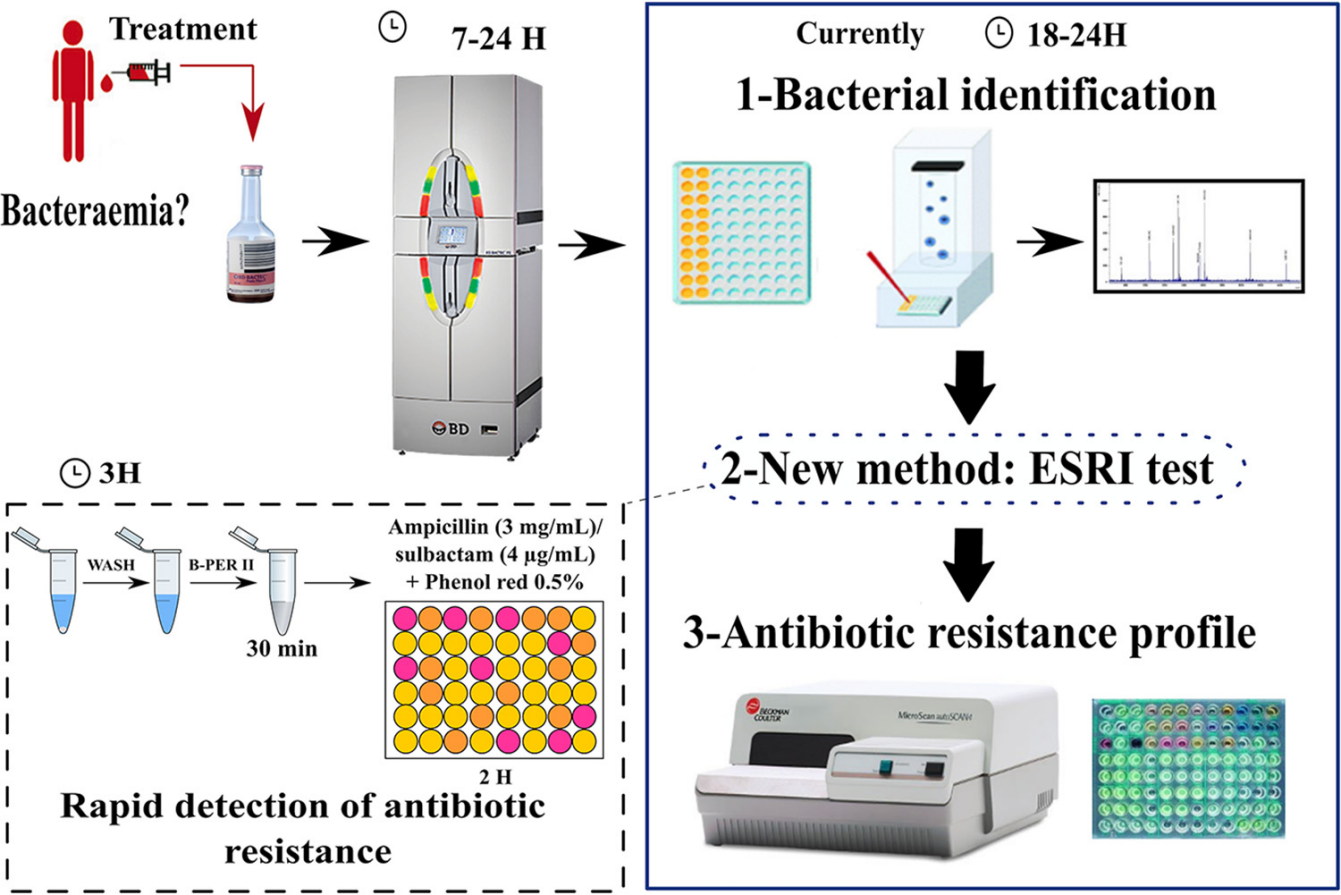
New detection method and its possible application in clinical practice. B-PER II, bacterial protein extraction reagent.

The ESRI test combines many advantages in relation to conventional clinical methods after positivity of a blood culture. It is semirapid, simple, and inexpensive. We demonstrated that the ESRI test allows us to differentiate with a high probability between isolates resistant to TZP (≤10 min of detection time) and ESRI developers (>10 to 120 min of detection time). These early detection times (less than 3 h) are an advantage compared with the 24 h needed by the conventional methods. We propose to use this test for all E. coli-positive blood cultures prior to the MIC testing (Fig. 3).

In addition, we would like to highlight that the ESRI test cannot be used to test resistance to SAM because SAM-susceptible isolates that developed ESRI (e.g., C1-174 isolate) and SAM-resistant isolates that did not develop ESRI (e.g., C1-74, C1-83, C1-138, and C2-12 isolates) were found. This discrepancy will limit the antimicrobial treatment options. We believe that the present ESRI test will overcome this limitation and allow differentiation between TZP-susceptible isolates and ESRI developer and TZP-resistant isolates where TZP should not be used for patient treatment.

The introduction of the ESRI test into clinical practice would have clinical implications. The rapid detection of ESRI in E. coli would be essential to establish an adequate antibiotic treatment for intra-abdominal infections, especially during the first 24 h, when the use of TZP is especially relevant. This might avoid a possible therapeutic failure due to the development of resistant bacteria, as well as an inappropriate use of antibiotics that in turn will contribute to increasing the rates of resistance of BL/BLI. Further clinical data are needed to assess the impact of the use of the ESRI test on the clinical prognosis of patients with intra-abdominal infections by E. coli. To this end, we need to determine the impact of the ESRI test on the duration of symptoms, clinical cure, complications of infection, need for subsequent surgical intervention, length of hospital stay, and mortality.

As limitations, the ESRI test was designed only for E. coli isolates growing in hemoculture bottles, unlike the above-mentioned tests that have also been tested with other genera of the *Enterobacterales* and from agar plates. However, it should be noted that E. coli is the main Gram-negative bacterium isolated from blood, peritoneal fluid, and bile samples (1), which are usually processed in hemoculture bottles in clinical microbiology laboratories. Future analyses of the ability of the ESRI test to detect TZP resistance in other bacterial genera and from different clinical samples are welcomed.

In conclusion, the ESRI test is a powerful weapon for the fight against antimicrobial resistance. This system may be an innovative contribution to microbiological diagnosis, allowing us to obtain information on the possible development of resistance to BL/BLI in less than 24 h and a better clinical prognosis, both in terms of survival and the absence of recurrence of resistant microorganisms, and also to achieve an economic impact associated with the improvement of the quality of health care.

## MATERIALS AND METHODS

### Bacterial isolates.

One hundred fourteen E. coli clinical isolates were obtained from bloodstream and intra-abdominal samples of patients with suspected bacteremia or intra-abdominal infections at the Hospital Universitario Virgen del Rocío, Seville (Spain). The E. coli ATCC 25922 strain was the control in all experiments. The study was approved by the Ethics Committee of the Hospital Universitario Virgen del Rocío of Seville (approval no. 0023-N-16).

### Antimicrobial susceptibility testing.

The SAM, AMC, and TZP antimicrobial susceptibility profiles were initially tested by broth microdilution using the MicroScan WalkAway NM44 panels (Beckman Coulter, Inc., USA). Isolate selection was made taking into account their BL/BLI susceptibility profiles (SSS, RSS, RRS, and RRR). The MICs of TZP were subsequently confirmed using the standard broth microdilution method (18). MICs were determined for the original isolates and isolates subjected to TZP pressure.

### Antimicrobial selection pressure.

The antimicrobial selection pressure experiment was performed as described previously (17). Overnight bacterial cultures grown in 10 ml of Mueller-Hinton broth (MHB) at 37°C were adjusted to 0.5 McFarland standard (10^8^ CFU/ml) and diluted to a final inoculum level of 10^5^ CFU/ml. The diluted inocula were incubated with subinhibitory concentrations of TZP (8:1 ratio) corresponding to 1-fold dilutions below the MICs at 37°C for 24 h. Positive bacterial growth was readjusted to 10^5^ CFU/ml, and adjusted cultures were incubated with a 2-fold-increased concentration of TZP. These steps were repeated until reaching a TZP concentration of 256/32 mg/liter or until a TZP concentration that did not allow bacterial growth was reached. At the end of the process, MICs were determined for all of the pressured isolates.

### Detection and sequencing of *bla_TEM_*, *bla_OXA-1_*, and *bla_SHV_* genes.

The *bla*_TEM_, *bla*_OXA-1_, and *bla*_SHV_ genes were analyzed in all studied isolates by PCR using the following primers: for *bla*_TEM_, forward, 5′-ATGAGTATTCAACATTTCCG, and reverse, 5′-CTGACAGTTACCAATGCTTA (17); for *bla*_OXA-1_, forward, 5′-GGATAAAACCCCCAAAGGAA-3′, and reverse, 5′-TGCACCAGTTTTTTTCCCATACA-3′ (26); and for *bla*_SHV_, forward, 5′-GGGTTATTCTTATTTGTCGC, and reverse, 5′-TTAGCGTTGCCAGTGCTC (27). All positive PCR products were screened by Sanger sequencing using an ABI 3500 genetic analyzer (Applied Biosystems, Foster, VA, USA) and analyzed using SnapGene Viewer 2.7.2 software and BLAST internet services (www.ncbi.nlm.nih.gov/BLAST).

### ESRI test.

The ESRI test is based on colorimetry- and pH-based detection of the hydrolysis of the β-lactam ring produced by the β-lactamase enzyme. The clinical isolates were inoculated into different blood culture vials (Bactec standard/10 aerobic/F and Bactec lytic/10 anerobic/F; Becton Dickinson, USA) at concentrations of 10^6^ and 10^2^ CFU/ml and incubated at 37°C with agitation at 180 rpm for 6 to 8 and 24 h in order to test the method’s efficacy in the time interval in which a patient blood culture with E. coli bacteremia is usually positive. After this time, 1.5 ml of the bacterial suspension grown in the blood culture was taken, transferred to an Eppendorf tube, and centrifuged at 9,600 × *g* for 2 min at room temperature (Thermo Fisher Scientific, USA). Subsequently, the pellet was resuspended with 1 ml of NaCl (0.9%) and centrifuged at 9,600 × *g* for 2 min at room temperature. Then, the pellet was resuspended in 100 μl of B-PER II lysis buffer (bacterial protein extraction reagent; Thermo Fisher Scientific, USA) and incubated at 37°C for 30 min. Afterwards, the sample was centrifuged again at 9,600 × *g* for 2 min at room temperature. Subsequently, 30 μl of the supernatant was added to a 195-μl solution consisting of 190 μl of ampicillin (3 mg/ml) (Sigma, Spain)-sulbactam (4 μg/ml) (Sigma, Spain) plus 5 μl of 0.5% (vol/vol) phenol red (Sigma, Spain) on a flat-bottom microplate. Finally, the plate was incubated at 37°C for 120 min, with readings every minute during the first 10 min and every 10 min for the rest of the time until 120 min (Fig. 1). In order to interpret the results, those samples with changes in color with respect to the negative control were considered positive results.

Negative test controls were performed in culture medium without the addition of ampicillin in order to check the stability of the color medium.

We should mention that, although the test is designed to detect TZP resistance and the possible development of ESRI, SAM was used in the test instead of TZP. This was for two reasons: (i) TZP resistance mediated by β-lactamases also leads to SAM resistance, and (ii) ESRI development always starts with SAM resistance, allowing us to detect SAM hydrolysis even if the isolate is susceptible to TZP and to avoid false-negative isolates.

### ESRI test validation.

The statistical parameters sensitivity, specificity, PPV, and NPV were used to determine the validity of the detection method developed and fine-tuned in this work. To calculate these parameters, we included the data for the isolates susceptible to TZP.

### Statistical analysis.

Descriptive analysis was performed for the times of positivity of the ESRI test, with the medians and interquartile ranges (IQR) being reported. Times of positivity of the ESRI test were analyzed using the Mann-Whitney U test. Differences were considered significant at a *P* value of <0.05. All statistical analyses were performed using SPSS software, version 23.0 (SPSS).
